# State of the Art and Perspectives on Polymer Science and Technology in China

**DOI:** 10.3390/polym18010136

**Published:** 2026-01-02

**Authors:** Sixun Zheng

**Affiliations:** Department of Polymer Science and Engineering, Shanghai Jiao Tong University, Shanghai 200240, China; szheng@sjtu.edu.cn

In 2023, the *Polymers* journal launched a Special Issue to report research by Chinese scholars focusing on the physics and processing of polymers, the development of new materials, and the application of new technologies. This Special Issue covers a small numbers of these rapidly accumulating, excellent contributions to the field of polymer science. Herein, the Guest Editors would like to present a few highlights from these investigations.

For the crystallization of polyolefins (e.g., PE), the introduction of a small number of comonomers would be subject to the melt memory effect, which is correlated with self-seeds, thus leading to an increase in the crystallization rate. The concentrations, distribution of comonomers, and melt temperatures can significantly affect the strength of the melt memory effect. Men et al. [1] investigated the crystallization behavior of a polyethylene random terpolymer with a small fraction of 1-octene and 1-hexene. After melting at various temperatures, the random terpolymer of PE displayed strong memory effects; the isothermal crystallization behavior revealed four melt temperature zones, at which the isothermal half crystallization time varied significantly. In the application of PE, the aging and degradation of polyethylene (PE) are inevitable. In elucidating the mechanisms and processes of the relevant chemical reactions, Lu and Yang et al. [2] investigated the distribution and evolution process of thermally oxidized products through molecular simulation. This work is essential for understanding the process in depth and for the development of anti-aging PE products.

Electrorheological fluid is an intelligent suspension in which particles are dispersed in a medium. Electrorheological fluid shows promise in applications such as dampening, biomorphic robotics, and microfluidic fields [3]. The rheological behavior of the suspension can be regulated with an external electric field. For most electrorheological fluids, the electric field-induced electrorheological response is quite weak, limiting practical applications. Recently, however, Yin et al. [4] investigated the effect of polyaniline nanoparticle morphologies on electrorheological behavior. By preparing the nanoparticles with spherical, fibrous, and lamellar morphologies, the authors investigated this behavior through rheological analysis and molecular dynamics simulation. It was found that electrorheological behavior can be enhanced through an increase in the morphological anisotropy of nanoparticles.

Polymeric foams have found wide-spread applications in a variety of fields, including aerospace, the automotive industry, and construction. Thermomechanical coupling in the molding process could incur compressive foam yields, reducing the performance of the foams. Recently, Liu et al. [5] investigated these issues with polymethacrylimide foams, providing insights into the impact of the sandwich structure, temperature, and pressure on the foam’s compression performance and failure mechanism. Chen and coworkers [6] reported the preparation of polyoxymethylene nanocomposites with molybdenum disulfide via a solid-state shear milling approach. Notably, the layers of molybdenum disulfide can be exfoliated via the strategy discussed, displaying a series of excellent thermomechanical properties.

Polyaniline is a promising organic cathode polymer for Li-ion batteries. However, the poor electrochemical and cycling instability limits its potential for practical applications. Qian et al. [7] reported the synthesis of polyaniline though chemical oxidative polymerization and found that the as-obtained materials had a highly reversible capacity. This is a facile method for improving the capacity of polyaniline. The upscaling of organic solar cells (OSCs) is a critical issue that hinders the commercialization of OSCs. Yang and Huang et al. [8] reported a nitrogen-blowing assisted method of fabricating a large-area organic solar module with the aid of high-boiling-point solvents, achieving a 15.6% power conversion efficiency. Notably, the utilization of high-boiling-point solvent generated a more uniform and smoother large-area film, whereas the assistance of nitrogen-blowing accelerates solvent evaporation, leading to an optimized phase-separated morphology.

Recently, considerable attention has been paid to flexible wearable electronic devices. Flexible strain sensors are typically composed of a soft matrix and conductive fillers. As a class of responsive materials, both rapid recovery and a small elastic modulus are required for the motion frequency of objects to be well matched. Polyurethanes are the optimal materials for flexible strain sensors. To obtain these two features simultaneously, Zhang et al. [9] reported an approach to fabricate a step-gradient polyurethane functional composite. It was found that the composite simultaneously displays both a fast recovery rate and a small elastic modulus.

To address the issues of sustainable development and environmental protection, developing biodegradable polymeric materials as alternatives to traditional petroleum-based materials has attracted considerable interest. By taking advantage of the biodegradability of polylactide, Wang and Ding [10] synthesized polyurethanes, the main chains of which were introduced with polylactide soft segments. In line with polylactide, the PUs displayed biodegradability. Simultaneously, the PUs also displayed improved thermomechanical properties. As the only natural source of aromatic biopolymers, lignin can be converted into value-added chemicals and biofuels, showing significant potential in realizing the development of green chemistry. Liu et al. [11] summarized the latest progress in the field of lignin depolymerization, including catalytic conversion through various thermochemical, chemo-catalytic, photocatalytic, electrocatalytic, and biological depolymerization approaches. Hemp fibers have been widely utilized in textile manufacturing and composite materials due to their breathability, specific mechanical strengths, and ultraviolet resistance. Bio-degumming is a promising alternative technology which could be used to generate hemp fibers in an eco-friendly manner. However, the lower efficiency of this approach has hindered its widespread adoption. Recently, Fu et al. [12] have demonstrated the impact of the composition and branching of polysaccharides on the bio-degumming of hemp roving by investigating the morphologies of hemp stem bast, hemp roving, and refined fibers. Preparing UV-curable resins with biomass has long been an objective. However, the existing UV-curable bio-based resins suffer from high viscosity and low mechanical strength. Yang et al. [13] have reported the synthesis of a soybean oil-based acrylate photosensitive resin, with epoxidized soybean oil being the raw material. Via a reaction with acrylic acid, the product displayed noticeably low viscosity. After being cured with UV, the soybean oil-based acrylate displayed excellent mechanical properties, namely, tensile strength and elongation at break.

## References

[1] Wang D., Li S., Lu Y., Wang J., Men Y. (2023). Men, Melt Memory Effect in Polyethylene Random Terpolymer with Small Amount of 1-Octene and 1-Hexene Co-Units: Non-Isothermal and Isothermal Investigations. Polymers.

[2] Zeng S., Lu D., Yang R. (2024). Effects of Crystallinity and Branched Chain on Thermal Degradation of Polyethylene: A SCC-DFTB Molecular Dynamics Study. Polymers.

[3] Hao T. (2001). Electrorheological fluids. Adv. Mater..

[4] Yuan J., Hu X., Zhao X., Yin Y. (2023). Electrorheological Effect of Suspensions of Polyaniline Nanoparticles with Different Morphologies. Polymers.

[5] Xing Z., Cen Q., Wang Q., Li L., Wang Z., Liu L. (2024). Compressive Mechanical Behavior and Corresponding Failure Mechanism of Polymethacrylimide Foam Induced by Thermo-Mechanical Coupling. Polymers.

[6] Feng S., Zhou X., Yang S., Tan J., Chen M., Chen Y., Zhang H., Zhu X., Wu S., Gu H. (2024). Preparation of Polyoxymethylene/Exfoliated Molybdenum Disulfide Nanocomposite through Solid-State Shear Milling. Polymers.

[7] Zhao R., Chang Z., Fu X., Xu M., Ai X., Qian J. (2024). Revisit of Polyaniline as a High-Capacity Organic Cathode Material for Li-Ion Batteries. Polymers.

[8] Cheng Y., Ji Y., Zhang D., Liu X., Xia Z., Liu X., Yang X., Huang W. (2024). Nitrogen-Blowing Assisted Strategy for Fabricating Large-Area Organic Solar Modules with an Efficiency of 15.6%. Polymers.

[9] Ning C., Gong D., Wu L., Chen W., Zhang C. (2024). Preparation of Gradient Polyurethane and Its Performance for Flexible Sensors. Polymers.

[10] Li X., Lin Y., Zhao C., Meng N., Bai Y., Wang X., Yu J., Ding B. (2024). Biodegradable Polyurethane Derived from Hydroxylated Polylactide with Superior Mechanical Properties. Polymers.

[11] Pei Z., Liu X., Chen J., Wang H., Li H. (2024). Research Progress on Lignin Depolymerization Strategies: A Review. Polymers.

[12] Yu T., Li P., Shu T., Liu T., Fu C., Yu L. (2024). Clarification of Bio-Degumming Enzymes Based on a Visual Analysis of the Hemp Roving Structure. Polymers.

[13] Chen Z., Wang S., Feng S., Huang Y., Hu Y., Yang Z. (2025). A Co-Blended and Compounded Photosensitive Resin with Improved Mechanical Properties and Thermal Stability for Nail Polish Application. Polymers.

